# Locating Structural Centers: A Density-Based Clustering Method for Community Detection

**DOI:** 10.1371/journal.pone.0169355

**Published:** 2017-01-03

**Authors:** Xiaofeng Wang, Gongshen Liu, Jianhua Li, Jan P. Nees

**Affiliations:** School of Electronic Information and Electrical Engineering, Shanghai Jiao Tong University, Shanghai, China; Brainnetome Center & The National Laboratory of Pattern Recognition, CHINA

## Abstract

Uncovering underlying community structures in complex networks has received considerable attention because of its importance in understanding structural attributes and group characteristics of networks. The algorithmic identification of such structures is a significant challenge. Local expanding methods have proven to be efficient and effective in community detection, but most methods are sensitive to initial seeds and built-in parameters. In this paper, we present a local expansion method by density-based clustering, which aims to uncover the intrinsic network communities by locating the structural centers of communities based on a proposed structural centrality. The structural centrality takes into account local density of nodes and relative distance between nodes. The proposed algorithm expands a community from the structural center to the border with a single local search procedure. The local expanding procedure follows a heuristic strategy as allowing it to find complete community structures. Moreover, it can identify different node roles (cores and outliers) in communities by defining a border region. The experiments involve both on real-world and artificial networks, and give a comparison view to evaluate the proposed method. The result of these experiments shows that the proposed method performs more efficiently with a comparative clustering performance than current state of the art methods.

## Introduction

The modern science of networks has brought significant advancements to our understanding of complex systems [1, 2]. One of the most important features for complex networks is community structure, which usually represent an organization of nodes in clusters, with high-density links within the clusters and comparatively low density between them. Such communities can be considered as independent compartments of networks. More importantly, community structures are often associated with organizational and functional characteristics of the underlying networks [3, 4]. Identifying communities helps uncover group characteristics and deduce their respective attributes, according to their role in the community, such as cores, and outliers. Therefore community detection is important in social network analysis, and is a significant tool that enables the study of mesoscopic structures.

Many community detection methods have been developed in recent years. These methods attempt to explore community structure characteristics in networks from various perspectives. The traditional graph partitioning methods divide the nodes into a predefined number of groups with predefined size, so that the inter-group edges is minimal [1]. Hierarchical clustering techniques reveal the multilevel grouping structure of a graph, which can be classified into agglomerative clustering and divisive clustering [2, 5]. Spectral clustering algorithms divide a network into groups by using the eigenvectors of similarity matrices [6, 7]. Modularity maximization technologies convert the task of community detection into an optimization problem of a modularity function to get optimal group partitioning [8–10]. In addition, fuzzy approaches also are used to compute communities, which quantify the strength of association between all pairs of nodes and communities with relax membership degree [11–13]. Most of the above methods are global approaches, but suffer from common limitations [14]. The main limitation is that global methods generally depend on prior knowledge of the entire network, such as the number of communities and network size, which are usually unavailable and unpredictable in advance, especially for large-scale and evolving networks. Moreover, many global methods with high accuracy tend to be computationally demanding. Therefore, it is non-trivial to get a good trade-off between accuracy and efficiency for community detection.

Many local approaches have been proposed to solve the limitations for uncovering communities listed above. They are based purely on local information of nodes. Local methods based on various optimizing strategies has been surveyed in recent study [14], where the local methods are empirically divided into clique-percolation based methods [15, 16], label propagation algorithms [17, 18], link clustering [19–21] and local expansion optimization methods [22–26]. Among them, local expansion optimization methods are widely used for local community detection in large networks, due to the advantages both in effectivity and accuracy. Such methods aim to optimize the local functions of community quality from the starting nodes [22, 23], while also being sensitive to initial seeds and built-in parameters. Lancichinetti *et al*. proposed a local optimization algorithm (LFM) based on a local fitness measure [24], which generates hierarchical community structure of the network by randomizing the starting node. The LFM may produce unstable results, due to its sensitivity to the starting nodes. Lee *et al*. introduced a greedy clique expansion (GCE) algorithm [25], which selects distinct cliques as starting seeds, and expands these seeds by greedy optimization. Huang *et al*. [26] introduced a similarity-based quality function and present a local tightness expansion algorithm (LTE) for revealing community structures from a random vertex. In addition, density-based clustering methods are also noteworthy. Xu *et al*. proposed a density-based clustering method extended from the DBSCAN algorithm to discovery community structures [27]. However, like other density-based clustering methods [28–31], it still depends on manual parameter choice and provides no automated way to find the appropriate parameters.

In this work, we present a new method for community detection which is termed as LCCD. It is a density-based clustering method, inspired by recent research on data analysis [32]where data points are clustered by finding the cluster centers. In order to investigate community structures in complex networks, we locate the structural centers in community structures by exploring the local centrality of nodes. Based on the assumption that cluster structural centers are characterized by a higher density than their neighbors and by a relatively large distance from nodes with higher densities, we propose a structural centrality to identify the local structural centers in networks. Then LCCD expands each community from the structural center to the boundary with a local search procedure. It is a fast and simple approach to identify intrinsic community structures, including cores and outliers. In addition, the local expansion method avoids the randomness of seed selection to improve its stability. Compared with previous local expanding algorithms that optimize the sub-graph quality from random seeds, the proposed method expands communities based on identified structural centers and accelerates the convergence to optimal solutions. Moreover, it avoids manual choice of built-in parameters.

The remainder of this paper is organized as follows. We present the related research about density based clustering methods for community detection in Section 2. Section 3 describes the formulation of community detection problem and the basic idea of LCCD algorithm. The proposed algorithm is described in detail in Section 4. Section 5 presents experimental results. Section 6 presents the conclusions.

## Related Work

Density-based clustering approaches have been commonly used in cluster analysis recently [33]. Density-based spatial clustering of application with noise (DBSCAN) is the pioneer work in this area [34]. In DBSCAN, the density is defined locally as the neighbors of a data point within a certain region. Given an appropriate density threshold *ϵ* and a minimum cluster size *μ*, one can assign regions of high density to different clusters and discard the points in regions with densities lower than this threshold as noise. The density-based clustering approach has been applied to social network analysis [27, 30]. Two algorithms DENGRAPH [30]and SCAN [27]extended from the DBSCAN have been proposed to detect communities, which introduce two different distance functions in the clustering process. DENGRAPH introduced an interaction-based distance that calculates the aggregated number of interactions between two users in a social network [30]. SCAN introduced structural similarity as the distance measure that calculates shared neighbors between nodes [27]. The SCAN can also identify hubs and outliers in a community. However, like DBSCAN, the two algorithms still depend overly on manually choosing thresholds, which can be difficult to determinate.

Other algorithms have been proposed for years. Huang *et al*. proposed a clustering algorithm gSkeletonClu by projecting a network to its core-connected maximal spanning tree [31]. It converts the density based rule to detect core connectivity components on a spanning tree. In this method, one of the two parameters is assumed given, and the other is regarded as index, and modularity is applied to choose the best partition. Huang *et al*. presented another density-based method called DenShrink that combines modularity optimization algorithm to overcome the resolution limit [35]. A physical topological distance was introduced in density-based clustering [36], which detects communities by additionally optimizing the kernel scale parameter. Because of the difficulty in setting appropriate parameters, Gong *et al*. [28]proposed to dispose all partitions under various parameters by classification, combination, decomposition and recombination, so as to produce proper community structures, but it increases computational cost and is unbeneficial for parameter selection. However, most of the above algorithms still depend on manual parameter choice and optimum iterative procedure, which limits their application in large-scale networks.

## Problem Formulations

Complex networks are generally represented as graphs with nodes and links between nodes. Such a representation has led to numerous insights on community structure. Due to the abundance of related works and the variety of adopted perspectives, there is no unique and widely accepted definition of community. Community definitions are formulated with reference to the network structure under study and are commonly bound to some property either of some set of vertices (local definitions) or of the whole network (global definitions) [37]. Local definitions focus on the concepts of subgroup cohesiveness and mutuality, such as cliques, k-cores. Global community conceptions consider community structure as a property of the whole network, such as normalized cut, conductance and modularity [38]. An alternative means of defining communities is by considering some community formation process, Such as label propagation scheme [17] and stochastic block modeling [39].

The basic assumption behind most local methods for community detection is that communities are essentially local structures, involving the nodes belonging to the groups plus extended neighbors of them [24]. Here, we present an alternative conception of community structure. From a mesoscopic perspective, we assume that a community can be regarded as a local centralized structure, which is naturally decomposed into a central node, cores and periphery. Central nodes should be well-connected to core nodes as well as peripheral nodes, although a network may not have an absolute center. Such structures can be commonly found in social networks, where nodes of some common attribute aggregate around the centers to form clusters. We call such central nodes as structural centers. Structural centers have not only high connectivity density in communities but relative large distance from each other. We therefore propose a new centrality based on the idea that structural centers are characterized by a higher density than their neighbors and by a relatively large distance from nodes with higher densities, in order to locate structural centers.

Exploring such structural centers of a network is important to community detection. Once the structural centers are identified, the number of clusters can be determined intuitionally. Moreover, it is able to overcome the randomness of seed choice for local expansion methods and has a faster convergence to optimal solutions. Although many centrality indices, including degree, betweenness, closeness and percolation centrality, have been defined to characterize the importance of nodes [40], these indices cannot characterize the centrality described above. There are two reasons for this issue. First, a centrality metric is optimal in one case but often sub-optimal in another case. Second, existing centrality indices are explicitly designed to produce a ranking which indicates the most important nodes [41], which cannot indicate the relative importance between nodes. In addition, a node with high centrality does not mean that it is the structural center of a community. For instance, nodes with high betweenness centrality are close to the boundary of community, and two nodes with high closeness centrality may be in the same community.

Based on above observation, we propose an alternative centrality, i.e., structural centrality, to measure such structural centers. It takes into account two indices: node density and relative distance in a two-dimensional space, which both depend on the distance measurement between nodes. We formulize relevant concepts and definitions on structural centrality as follows. Generally, an undirected and unweighted network employs the graph notation *G* = (*V*, *E*), where *G* represents the whole network, *V* stands for the set of all nodes and *E* for the set of all edges. The topologic structure of the network can be represented by an adjacent matrix *A*, which is crisp relation to characterize the connectivity among nodes with 0 or 1.

**Definition 1**. *(Node Density) Let A be the adjacency matrix of network*. *The density of node i in the network is defined as*:

$$\begin{array}{c} \rho_{i}=\sum_{j}\psi (d_{ij}-d_{c}), \end{array}$$ (1)

*where ψ*(*x*) = 1 *if x* ≤ 0, *and ψ*(*x*) = 0 *otherwise; d*_*ij*_ *denotes the distance between node i and j in A*, *and d*_*c*_ *is a cutoff distance*.

In this definition, *ρ*_*i*_ is equivalent to the number of neighbor nodes within the distance *d*_*c*_. Geodesic distance is used to measure the distance between nodes, other distance metrics, such as common neighbors, information distance, can be used as alternative measures. The algorithm is sensitive only to the relative magnitude of *ρ*_*i*_, which implies that the results are robust with respect to choice of *d*_*c*_. Based on the suggestion in the reference [32], the cutoff distance *d*_*c*_ can be automatically chosen so that the mean number of neighbors is around 1 to 2 percent of the total number of the nodes in the network. In our experiments, we observe that *d*_*c*_ is restricted to several values which are smaller than the network diameter. In most case *d*_*c*_ equals 1, this issue is discussed in appendix. Moreover, varying cutoff distance *d*_*c*_ on a variety of networks produces mutual consistent results, which verifies that the results are robust to the choice of *d*_*c*_. Therefore, as a rule of thumb, we set *d*_*c*_ = 1 in our method for community detection. In this case, the node density is equivalent to degree centrality.

**Definition 2**. *(Relative Distance) The relative distance δ*_*i*_ *is measured by computing the minimum distance between the node i and any other nodes with higher density*, *it is formulized as follows*:

$$\begin{array}{c} \delta_{i}=\min_{j:\rho_{j}>\rho_{i}}\left(d_{ij}\right), \end{array}$$ (2)

For the node with the largest local density, we conventionally take *δ*_*i*_ = max_*j*_(*d*_*ij*_). Note that *δ*_*i*_ is much larger than the nearest neighbor distance, but only for nodes that are local or global maxima in the density. So, community structural centers are recognized as nodes for which the value of *δ*_*i*_ is anomalously large.

**Definition 3**. *(Structural Centrality) The structural centers are characterized by a higher density than their neighbors and by a relatively large distance from nodes with higher density*. *The structural centrality of node i is defined as*:

$$\begin{array}{c} {\text{sc}}_{i}=\rho_{i}*\delta_{i}, \end{array}$$ (3)

From this definition, the structural centrality is proportional to node density and relative distance, respectively. Eq (3) expresses a simple and intuitive form of the definition. The value of structural centrality can be normalized with different formulations during the computation. Relative distance measures the distance between nodes with density maxima, so it avoids the situation that more than one node with high centrality in the same community are identified as the structural centers. Thus, structural centers of clusters can be recognized as these nodes with the local maxima of the structural centrality. Moreover, the structural centers are obviously separated from other nodes in the plot of relative distance as a function of node density.

**Fig 1:**
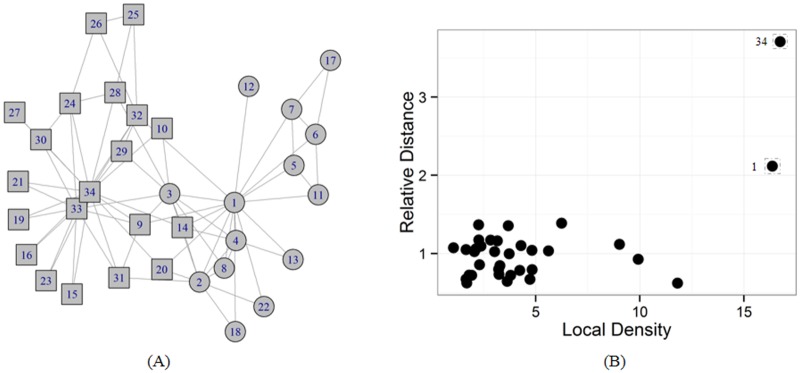
A schematic example to illustrate the idea of our method. (A)The Zachary’s karate club network with two clusters. (B)The decision graph for the nodes in the network.

This observation is illustrated in Fig 1 by the Zachary’s karate club network [42]that is a real-world social network. This interactive network with 34 nodes, ultimately split into two distinct groups, because of a disagreement between the administrator (vertex 1) and the instructor (vertex 34), as shown in Fig 1(A). Fig 1(B) shows the plot of *δ*_*i*_ as a function of *ρ*_*i*_ for each node. We can find that node 1 and node 34 have the density maxima which are identified as the structural centers. The result is consistent with the description of the original network. The representation of the plot is called decision graph, where a slight jitter is imposed on their actual values to avoid the overlap between nodes. In fact, the node 34 has the maximal density *ρ*_34_ = 17 and maximal relative distance *δ*_34_ = 4, and node 1 has *ρ*_1_ = 16 and *δ*_1_ = 2, while the rest nodes have lower density with the same relative distance *δ* = 1. Especially, the nodes 4 and node 32 have same values of *ρ* and *δ*, while they belong to different clusters because they are close to different structural centers. As expected, the nodes with both high local density and high relative distance are identified as the community structural centers.

The identified structural centers are separated distinctly from other nodes in decision graphs so that the structural centers arise automatically, which is especially obvious in networks with heterogeneous degree distributions. By a local expansion around the structural centers, underlying community structures can be uncovered. In addition, we can choose the exact number of structural centers by the plot of structural centrality sorted in decreasing order as a function of node number. This graph shows that this quantity is by definition large for structural centers. Although it’s possible that there are more than two nodes with the same largest structural centrality in one community, especially for a symmetric graph such as complete graph, the proposed method can also obtain stable results. This is because that the proposed method is a deterministic algorithm. On one hand, the algorithm selects the first node as structural center and eliminates the nodes close to the structural center in the procedure of locating structural center. So it can identify unique structural center for each community. On the other hand, during the expansion, the algorithm chooses the structural center in decreasing order and neglects other nodes if they have been identified in one community, and ultimately get a unique community.

## Materials and Methods

In this section, we present our algorithm framework for uncovering underlying community structures in unweighted and undirected networks. Our method is inspired by density-based clustering formulation proposed by Rodriguez and Laio in cluster analysis [32]. However, our method focuses on node clustering in complex networks and uncover underlying community structures. We propose the LCCD algorithm based on the idea that structural centers are surrounded by neighbors with lower local density and they are at a relative large distance from any nodes with a higher local density. This idea forms the basis of a clustering procedure in which the number of communities arises intuitively, disjoint communities are detected, cores and outliers are automatically identified by node assignment. The LCCD algorithm is divided into three phases: location of community structural centers (LCC algorithm), local expansion (LCE algorithm) and node assignment (NA algorithm).

Given an unweighted and undirected network *G* = (*V*, *E*), where *V* represents the set of nodes and *E* represents the set of edges. A community partition of the network is represented as *P* = {*C*_1_, *C*_2_,⋯, *C*_*K*_}, where *C*_*i*_ stands for the *i*th community structure in the partition and *k* stands for the number of communities. The main procedure of the LCCD algorithm is given as Algorithm 1. Firstly we present the approach for locating community centers with the proposed structural centrality, which is the core of the method. Secondly, a local expansion clustering algorithm based the identified structural centers is proposed to find the optimal clustering of network nodes. Finally, LCCD performs a node assignment process by finding a border region to identify cores and outliers in communities.

**Algorithm 1** Main procedure of Local Expanding Algorithm (LCCD)

**Input**: Adjacency matrix *A* of network *G* = *(V, E)*.

**Output**: Network community partition *P* = *{C*_*1*_, *C*_*2*_ ,…,*C*_*k*_}, and set of cores and outliers for each community.

1: Label all nodes in *V* unclassified U;

2: Locate the structural centers by calling LCC algorithm;

3: Take a structural center as the initial community, and expand local communities by calling LCE algorithm;

4: Identify the cores and outliers by calling NA algorithm;

5: Update U by removing the identified nodes from U, repeat 3 and 4 until all nodes have been grouped;

6: **return** Network partition *P*, cores and outliers for each community.

### Locating Community Structural Centers

As described above, community structural centers are characterized by a higher density than their neighbors and by a relatively large distance from nodes with higher densities. In order to locate the structural centers in a network, we define a structural centrality. The structural centrality not only measures the local centrality of node but also quantifies the interrelation between clusters. The structural centers of community structures are recognized as the nodes with local maxima in the structural centrality of network nodes. In the plot of the structural centralities, this quantity for structural centers is obviously large, so that the structural centers are automatically separated from other nodes. This process of locating structural centers can identify a unique structural center for each community, which implies that the number of communities arises intuitively.

**Fig 2:**
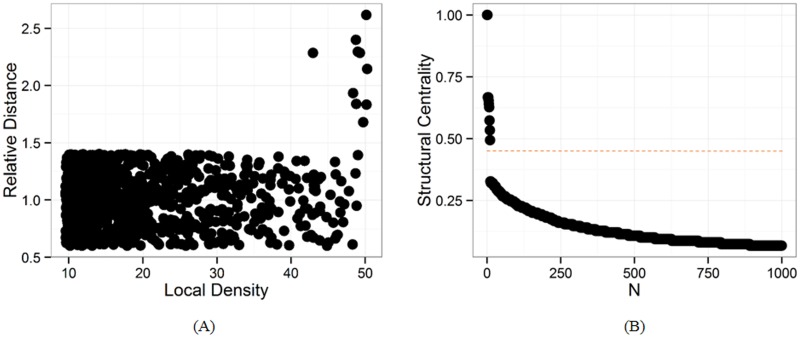
Location of structural centers on synthetic LFR network with 1,000 nodes and 9 ground-truth communities. (A)The node distribution in the decision graph. (B)The plot of structural centrality sorted in decreasing order as a function of node number for the network.

Fig 2 illustrates the procedure of locating the structural centers on a LFR benchmark network [43]which have similar properties found in real networks. We generate a benchmark network with 1,000 nodes and 9 known community structures. Fig 2(A) shows the node distribution in the decision graph. In the decision graph, we observe only 9 nodes which are distinctly separated from others. These nodes have local maxima in node density and relative distance and are identified as the underlying structural centers. However, it is still not clear to locate the exact number of structural centers. Through observation on the distribution of structural centrality, a hint for choosing the number of structural centers is provided by the plot of structural centers sorted in decreasing order (Fig 2(B)). The figure shows that the structural centrality is by definition large for structural centers and gap away from other nodes. Therefore, these nodes above the horizontal dash line correspond to the 9 structural centers.

This observation provides the basis for a criterion for the choice of the structural centers. In order to identify the structural centers exactly, we propose to select the structural centers with a threshold. This threshold can be chosen by the plot of structural center sorted in decreasing order. These nodes with structural centralities above this threshold are identified as underlying structural centers. In some cases, however, a low threshold may lead to some general nodes being selected. For validating the reliability of selected structural centers, we further check if there is a candidate node that is close to the identified structural centers. As defined above, structural centers have large relative distance from each other, and only the nodes corresponding to structural centers are separated by a sizeable gap from the other nodes.

The main steps of LCC algorithm for locating the community structural centers are given as Algorithm 2. The set of structural centers is represented with $C=\{c_{1},c_{2},\ldots ,c_{k}\}$, where *k* denotes the number of communities. LCC first calculates the structural centrality of each node in network and draws the plot of structural centrality sorted in decreasing order. By a given threshold *ε*, LCC then selects these nodes with higher centrality than the threshold *ε* as candidate structural centers (*cc*) and takes the first node as the first cluster center. If the distance between the second node and the first structural center is not less than the cutoff distance *d*_*c*_, the second candidate node will be chosen as the next structural center, otherwise, it will be deleted from the candidate queue. This process is repeated until all structural centers are located. It means that the nodes close to structural centers are eliminated from the candidate queue by this procedure.

**Algorithm 2** Locating Community Centers (LCC)

**Input**: Adjacency matrix *A* of network *G* = *(V, E)*, threshold *ε*.

**Output**: Community structural centers $C=\{c_{1},c_{2},\ldots ,c_{k}\}$.

1: //Calculate the structural centrality distribution of nodes;

2: *sc*_*i*_ ← *ρ*_*i*_ * *δ*_*i*_;

3: //Insert candidate structural centers into queue *cc*

4: *cc* ← {*v*_*i*_ ∣ *sc*_*i*_ > *ε*, *v*_*i*_ ∈ *V*};

5: *cc* ← sort *cc* in descending order;

6: //Check whether *v* is a community structural center;

7: *k* ← 0;

8: **while** *cc* ≠ ∅ **do**

9:  *k* ← *k* + 1

10:  *c*_*k*_ ← the first node in *cc*

11:  remove *c*_*k*_ from *cc*

12:  //Merge close candidate centers;

13:  **for** each node *v* ∈ *cc* **do**

14:   **if** *dist*(*v*, *c*_*k*_) < *d*_*c*_ **then**

15:    remove v form cc

16:   **else**

17:    Next

18:   **end if**

19:  **end for**

20: **end while**

21: **return**C.

The results of the LCC algorithm do not depend on the order of nodes, because this algorithm ranks nodes by their structural centrality, and searches nodes in descending order. Moreover, the structural centrality of a node does not need recalculation. The structural centers have a higher density than their neighbors and a relatively large distance from nodes with higher densities, which implies the only node with the largest structural centrality is identified as the structural center in one community. Therefore, the number of structural centers indicates the number of communities in a network. The LCC algorithm can also be applied to other community detection methods, especially for these methods that need to be given the number of clusters manually in community detection.

### Local Community Expansion

After identifying community structural centers, we can obtain corresponding community structures around these structural centers based on a measure of the sub-graph, which is similar to other community expanding methods that expand a community based on a seed node, such as LFM [24], GCE [25] and LTE [26]. However, there are some significant differences. Firstly, communities expand locally around the structural centers in the proposed method, in contrast with other clustering algorithms where seed nodes are selected randomly. Moreover, such expanding strategy accelerates the convergence to optimal solutions. Here, we define a new local community measure, i.e., sub-graph density. A community is recognized as the sub-graph identified by maximization of its density measure.

**Definition 4**. *(Subgraph Density) For a community C with n*_*C*_ *nodes and m*_*C*_ *edges*, *the sub-graph density is defined as follow*:

$$\begin{array}{c} D_{C}=\frac{m_{C}}{\sum_{i=1}^{n_{c}}\rho_{i}}, \end{array}$$ (4)

*where ρ has been defined in* Eq (1). *It is equivalent to* $D_{C}=m_{C}/\sum_{i=1}^{n_{c}}k_{i}$ *when d*_*c*_ = 1, *where k*_*i*_ *is the degree of node i*.

In our local expansion, we adopt a greedy strategy which aims to find a sub-graph starting from a structural center such that the inclusion of a new node would increase the sub-graph density *D*_*C*_, or the elimination of a node from the sub-graph would lower the sub-graph density *D*_*C*_. Thus, we can get a complete community based on a structural center by maximization of its sub-graph density. The similar idea of uncovering communities by a local optimization of some metric has already been applied in earlier work [22–24, 26]. The expansion procedure based on the LCC is given in Algorithm 3.

**Algorithm 3** Local Community Expanding Algorithm (LCE)

**Input**: Adjacency matrix *A*, a Community structural center c∈C.

**Output**: Community structure *C*.

1: Label all unclassified nodes in *V* as *U*

2: **while** *U* ≠ ∅ **do**

3:  initialize community *C* ← ∅

4:  **if** *c* ∈ *U* **then**

5:   insert c into *C*

6:  **end if**

7:  insert neighbors of community *C* into *Q*

8:  **while** *Q* ≠ ∅ **do**

9:   **for** each node *v* in *Q* **do**

10:    Δ*D*_*v*_ ← *D*_*C*+*v*_ − *D*_*C*_

11:    *m* ← *sort*(Δ*D*_*v*_)

12:   **end for**

13:   **if** *m* < 0 **then**

14:    break

15:   **end if**

16:   add *v* into *C*

17:   update neighbors set *Q* of community *C*

18:  **end while**

19:  update unclassified node set *U*.

20: **end while**

21: **return** *C*.

The algorithm performs a local expansion from center to boundary. A natural community is identified by the maximization of sub-graph density. The community around structural center *c* can be uncovered with the following procedure. To begin with, the structural center *c* is chosen as the initial community *C*, where *D*_*C*_ = 0. Then, we consider the neighbors of community *C* not included in *C* and evaluate the gain of sub-graph density that would take place by adding neighbors into *C*. A neighbor node is added into the community for which the gain is maximum, but only if the gain is positive. If a node turns out to have negative gain, it is removed from *C*. This process is repeated iteratively until no further improvement is achieved and a local maxima of sub-graph density is attained. Finally, all community structures corresponding to identified structural centers can be detected by this local expansion procedure.

### Node Assignment

In this section, we further explore different roles of nodes in a community during node clustering, based on attained community structures. Except for structural centers in communities, there are two types of nodes that play special roles: core nodes that cohesively connected to the structural center and outliers that are marginally connected to communities [27]. Identifying cores is useful because they compose the principal part of community structures. In addition, outliers may play a special role in community structures. We attempt to identify the cores and outliers in a community by defining a border region in the community. Some definitions are formulized as follows.

**Definition 5**. *(Community Border) In a community C*_*i*_, *a node v* ∈ *C*_*i*_ *is called a border node*, *if there exists a node w* ∉ *C*_*i*_ *within a cutoff distance d*_*c*_ *of v*. *All border nodes in community C*_*i*_ *consist of a community border region denoted by* $B_{i}$:

$$\begin{array}{c} B_{i}=\left\{v|v\in C_{i},w\notin C_{i},dist\left(v,w\right)\leq d_{c}\right\}, \end{array}$$ (5)

**Definition 6**. *(Border Density) In a community structure C*, *the highest density within its border region* B∈C *is defined as the border density of C denoted by ρ*_*b*_:

$$\begin{array}{c} \rho_{b}=\max_{i\in B}\rho_{i}, \end{array}$$ (6)

**Definition 7**. *(Core) In a community structure C*, *a node v* ∈ *C is called the core node of community*, *if its density is higher than ρ*_*b*_. *Cores*(*C*) *denotes the set of cores in community C*, *formally*:

$$\begin{array}{c} Cores\left(C\right)=\left\{v|\rho_{v}\geq \rho_{b},v\in C\right\}, \end{array}$$ (7)

**Definition 8**. *(Outlier) In a given community structure C*, *a node v* ∈ *C is called an outlier*, *if its density is lower than ρ*_*b*_. *Outliers*(*C*) *denotes the set of outliers in community C*, *formally*:

$$\begin{array}{c} Outliers\left(C\right)=\left\{v|\rho_{v}<\rho_{b},v\in C\right\}, \end{array}$$ (8)

In some density-based methods like SCAN [27], one consider core nodes with density above a threshold, this might lead to low density communities being classified as outliers. In our work, we characterize different roles of nodes in a community by finding a border region with their density. The border region is defined as set of nodes assigned to the community but being within a distance *d*_*c*_ from these nodes belonging to other communities. We can find a border region for each community. In a community, we find the node with highest density within its border region, and take the highest density as border density *ρ*_*b*_. Therefore, nodes in the community whose density is higher than *ρ*_*b*_ are considered as community cores, and the other nodes are identified as outliers.

**Fig 3:**
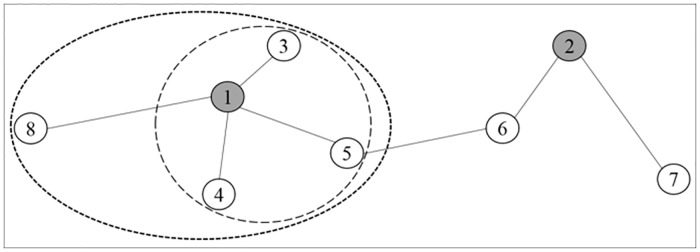
An illustration of node assignment with an abstract graph.

As shown in Fig 3, nodes are labeled with their density order, and the length of edges denotes the distance between nodes. Node 1 and 2 are identified aa structural centers in two communities, respectively. In community 1 (ellipse region), node 5 which is the nearest to the other community, is of the highest density in border region (circular region), so *ρ*_*b*_ = *ρ*_5_. According to our assignment criterion, we can identify node 3 and 4 as cores and node 8 as outlier.

Based on the above description, the main steps of the proposed node assignment algorithm are given in Algorithm 4. This algorithm identifies cores and outliers by finding a border region for each community, based on detected network partition. At the beginning, for each community structure, we identify the neighbors of each internal node within a trade-off distance *d*_*c*_. If there is a neighbor that belongs to other community, this internal node is identified as a border node, and the border density *ρ*_*b*_ takes the average density of the two nodes. Then, assignment algorithm finds all border nodes to form a border region, and update the border density with the maximal *ρ*_*b*_. A node with higher density than *ρ*_*b*_ is identified as cores, otherwise, it is an outlier. The algorithm does not stop until all nodes have been assigned to one of these roles.

**Algorithm 4** Node Assignment Algorithm (NA)

**Input**: Adjacency matrix *A*, a Community structure *C*.

**Output**: Cores and outliers in *C*.

1: initialize $\rho_{b}^{C}\leftarrow 0$

2: **for** each node *v* ∈ *C* **do**

3:  *N*_*d*_*c*__(*v*) ← neighbors of *v* within distance *d*_*c*_

4:  **for** each node *w* ∈ *N*_*d*_*c*__(*v*) **do**

5:   **if** *dist*(*v*, *w*) ≤ *d*_*c*_ **then**

6:    $\bar{\rho }\leftarrow \left(\rho_{v}+\rho_{w}\right)/2$

7:    **if** $\bar{\rho }>\rho_{b}^{C}$ **then**

8:     $\rho_{b}^{C}\leftarrow \bar{\rho }$

9:    **end if**

10:   **end if**

11:  **end for**

12:  **if** $\rho_{v}\geq \rho_{b}^{C}$ **then**

13:   label *v* as a core

14:  **else**

15:   label *v* as a outlier

16:  **end if**

17: **end for**

18: **return** *Cores*(*C*) and *Outliers*(*C*).

### Complexity Analysis

In this section, the computational complexity of the proposed algorithm LCCD is analyzed. As described above, LCCD algorithm includes three phases, locating structural centers (LCC), local community expanding process (LCE), and node assignment process (NA). Given a network with *n* nodes and *m* edges, the time complexity for computing structural centrality distribution scales as *O*(*n*^2^), and the search process of structural centers requires *O*(*k*log*n*) time, where *k* denotes the number of community structures, so the time complexity of the LCC algorithm scales as *O*(*n*^2^ + *k*log*n*). In the expanding phase, the local expanding process depends on the number of community structures, the complexity of a single community expansion is linear on typical and sparse networks. So the time complexity for the local expanding algorithm LCE scales as *O*(*kn*). The node assignment process relies on community size. If we assume that the average size of network communities is s, the NA algorithm cost *O*(*s*(*n*−*s*)) time. The time complexity for NA algorithm scales as O(sn). So the total time complexity of the LCCD algorithm scales as *O*(*n*^2^ + *kn* + *sn*). For a community structure in a network, its size is far smaller than the scale of the network. Therefore, the time complexity of LCCD can be simplified as *O*(*n*^2^).

For the compared algorithms mentioned in Section 5, we have collected estimates of how the cost scales with network observables. For general graphs irrespective of density, the Walktrap algorithm has the highest computational complexity *O*(*n*^4^), and the time complexity of Infomap scales as *O*(*n*^2^log*n*), while the efficient modularity-based algorithm Louvain has lower time complexity which scales as *O*(*n*^2^). The time complexity of the CNM algorithm scales as *O*(*n*^2^ *d*log*n*) where d denotes the depth of the dendrogram. LPA takes a near linear time, while it is essentially an indeterministic algorithm that needs multiple iterations to attain stable performance. In general, the most accurate method tends to be more computationally expensive [44]. Compare with these algorithms, the proposed algorithm LCCD has lower time complexity and higher accuracy, this will be shown in following experiments.

## Experimental Results

In this section, we test the performance of the LCCD algorithm against a variety of networks that have been commonly used in community detection. We compare LCCD with various classic community detection algorithms on real-world networks and synthetic networks to illustrate the performance of the proposed method on uncovering community structures. In addition, we adopt two important evaluation criteria, i.e., modularity and normalized mutual information, to evaluate the effectiveness of community detection algorithms. To ensure the stability of results, all algorithms have been independently run 10 times on each dataset. Our algorithm is implemented in RStudio, and all the experiments were conducted on a PC with a 2.0GHz Intel processor and 4 GB of RAM.

### Evaluation Criteria

To evaluate the effectiveness of a community detection algorithm in the experiments, we introduce two different criteria. In real world networks of various size and levels of community cohesiveness, there is no ground truth community structure. Therefore, we adopt modularity to measure the quality of division into communities and the cohesiveness of discovered community structures. On the other hand, in order to measure the similarity between the planted partition and that uncovered by algorithms, we adopt the normalized mutual information (NMI) to evaluate the performance of our method against synthetic networks of known ground truth community structures.

The modularity is one of the most popular criteria for measuring the quality of community partitions [3]. It is based on the idea that a random graph is not expected to have a community structure, so the possible existence of communities is uncovered by the comparison between the actual network partition and corresponding null model [1]. Modularity can be written as follows:

$$\begin{array}{c} Q=\frac{1}{2M}\sum_{ij}\left[A_{ij}-P_{ij}\right]\delta \left(C_{i},C_{j}\right), \end{array}$$ (9)

where *M* denotes the total number of edges in a network, *A*_*ij*_ represents the connection relation between node *i* and node *j* in adjacency matrix, *P*_*ij*_ represents the expected number of edges between the node *i* and node *j* in the null model. A standard choice is *P*_*ij*_ = *k*_*i*_ *k*_*j*_/2*M*, *k*_*i*_ and *k*_*j*_ being the degree of node *i* and node *j*. *C*_*i*_ represents the community that node *i* belongs to. if node *i* and node *j* belong to the same community, the value of *δ*(*C*_*i*_, *C*_*j*_) equals one, zero otherwise.

Normalized mutual information (NMI) is an information-theory based measurement, which is widely used in measuring the performance of graph clustering algorithms [44]. It enables one to compare partitions and covers, the measurement NMI can be defined as:

$$\begin{array}{c} NMI\left(A,B\right)=\frac{-2\sum_{i=1}^{C_{A}}\sum_{j=1}^{C_{B}}\log\frac{N_{ij}N}{N_{i.}N_{.j}}}{\sum_{i=1}^{C_{A}}N_{i.}\log\frac{N_{i.}}{N}+\sum_{j=1}^{C_{B}}N_{.j}\log\frac{N_{.j}}{N}}, \end{array}$$ (10)

where *A* and *B* denotes detected community partition and real partition respectively, and *C*_*A*_, *C*_*B*_ is the number of communities in *A* and *B*. *N* is the confusion matrix, *N*_*ij*_ is the number of nodes in common between community *C*_*i*_ and *C*_*j*_, *N*_*i*._ is the sum over row *i* of *N* and *N*_.*j*_ is the sum over column *j* of *N*. Note that the value of NMI ranges between 0 and 1, higher values mean more accurate results for an algorithm.

### Comparison with Other Methods

In this section, the performance of the proposed algorithm LCCD for uncovering community structures is illustrated by comparing with some widely used algorithms. The compared algorithms contain three local methods and three global methods. The local community detection methods include LPA [25], SCAN [27] and Walktrap [45]. LPA represents a label propagation algorithm that is based on an iterative dynamic processes. It is computationally efficient and conceptually simple for identifying network communities. SCAN is a density-based clustering method, which is effective to discovery sub-graph structures with well-specified properties. Walktrap algorithm computes communities using random walk, which is excellent for vertex partition. The global algorithms include two representative modularity-based methods: CNM [8] and Louvain [46], and an information-theory based method called Infomap [47]. CNM is a classic modularity optimization algorithm for community detection. Louvain also optimize modularity but with a heuristic search process. The Infomap algorithm turned out to be the best performing algorithms in community detection and has remarkable performance [48]. All of the above algorithms are used to evaluate and compare the performance of methods in community detection. In our experiments, all algorithms are tested on a variety of synthetic networks and real-world networks.

#### Test on Synthetic Networks

In the following, we will evaluate the clustering accuracy of LCCD, which is compared with other classic algorithms on computer-generated networks, including GN benchmark networks [42] and LFR benchmark networks [43]. By varying the parameters of benchmark graphs, we generate a variety of benchmark graphs. We adopt the NMI measure to evaluate the accuracy of algorithms on these benchmarks. In order to avoid the randomness of benchmark networks, we generate 10 networks with the same parameters and take the average as the final result.

#### GN benchmark networks

We first test all algorithms on the GN benchmark networks, which have well defined community structures [42]. We generate a variety of benchmark graphs with various parameters, each graph is constructed with 128 nodes that are divided into four clusters with 32 nodes. Edges between node pairs are placed randomly, with probability *P*_*in*_ for nodes belonging to the same community and *P*_*out*_ for nodes in different communities, where *P*_*out*_ < *P*_*in*_. The probabilities are adjusted to keep the average degree *z* of each node to 16, i.e., *z*_*in*_ + *z*_*out*_ = 16, where *z*_*out*_ implies the external degree of a node. For simplicity, we use mixing parameter *μ* ranging from 0.1 to 0.8 to represent the average ratio of external degree *z*_*out*_ in total degree for each node. The greater the mixing parameter, the more difficult to uncover the community structure in graphs.

**Fig 4:**
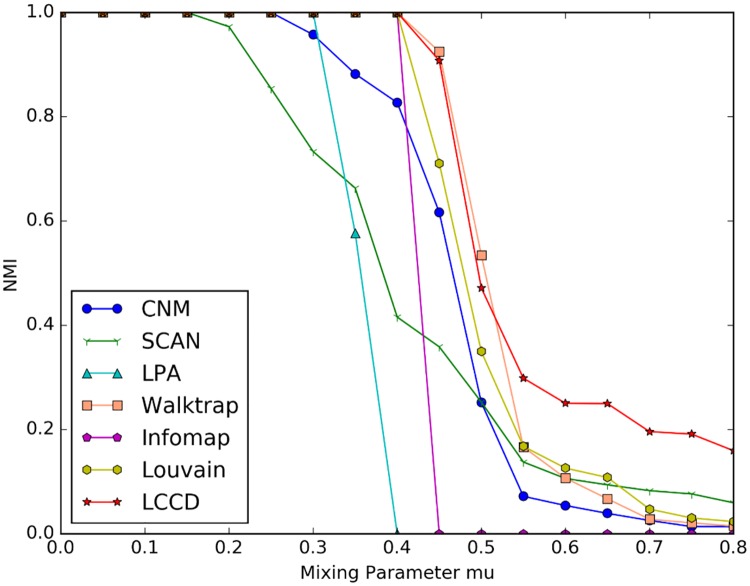
Comparison on GN benchmarks. The NMI value is averaged over 10 networks generated with the same parameters.

Fig 4 shows the experimental results for different algorithms on GN benchmark networks. As shown in the figure, all algorithms get NMI = 1 when the mixing parameters *μ* is less than 0.15, this means that all algorithms can identify the true community structures. However, the performance of these algorithms decline to various degree, as the mixing parameter increases. When *μ* is no larger than 0.4, there are only four algorithm, i.e., Walktrap, Infomap, Louvain and LCCD, can uncover the ground true community structures. As the mixing parameter further increase, the border between communities becomes more obscure, and the accuracy of algorithms goes down greatly. As can be seen from the figure, however, the LCCD still get greater NMI values when *μ* > 0.4, compared with other algorithms. The NMI scores of LCCD are slightly lower than that of Walktrap but higher than that of Louvain when *μ* is no less than 0.4 and no greater than 0.55, but LCCD obviously superior to Louvain when *μ* > 0.55 on GN benchmark networks. This is because LCCD adopts a local clustering process and is effective in avoiding the resolution limit of modularity [9], while the Louvain algorithm tends to produce some big communities by merging small communities. Moreover, the Walktrap algorithm gains remarkable results when *μ* < = 0.55. In fact, Walktrap makes use of a local random-walk based similarity between nodes to derive an optimal clustering structures. This further demonstrates the advantage of local method in community detection.

#### LFR benchmark networks

For a more standardized comparison, we also test LCCD algorithm on the LFR benchmark networks [43], to evaluate its performance. The LFR networks reflect the heterogeneity in the distribution of node degree and community size, which are claimed to possess properties found in real networks. There are some important parameters for the benchmark networks: *n*: number of nodes; *k*: average degree of the nodes; *maxk*: maximum degree; *minc*: minimum for the community sizes; *maxc*: maximum for the community sizes; *t*_1_: exponent for the degree distribution; *t*_2_: exponent for the community size distribution; *μ*: mixing parameter. The mixing parameter *μ* means that each node shares the fraction *μ* of neighbors with other nodes within the community and connect a fraction (1−*μ*) nodes without the community. By varying these parameters, we generate benchmark networks with different community structures. For a benchmark graph, the higher the mixing parameter it has, the more difficult it is to reveal the community structure.

To analyze the performance of the algorithm, and check how much the performance of the algorithm is affected by the network scale and community size, here, we generate a variety of unweighted and undirected benchmark networks with two kinds of network scale, *N* = 1,000 and *N* = 10,000. For each scale, two kinds of networks are generated with different ranges of community size, where *S* means that the sizes of communities in the network are relatively small and *B* means that the sizes of communities are relatively large. All network are generated with fixed value *t*_1_ = 2 and *t*_2_ = 1. The other parameters of these benchmark networks are given in Table 1. We generate various non-overlapped networks for each type dataset by ranging mixing parameter *μ* from 0 to 0.8 with an interval of 0.05.

**Table 1:** The main parameters of the generated benchmark networks.

Network	*N*	〈*k*〉	*maxk*	*minc*	*maxc*
1000*S*	1,000	20	50	10	50
1000*B*	1,000	20	50	20	100
10000*S*	10,000	40	100	50	100
10000*B*	10,000	40	100	100	200

*N* represents number of nodes; 〈*k*〉 denotes average degree of nodes; *maxk* represents the maximum degree of nodes; *minc* denotes the minimum community size, and *maxc* the maximum one. All benchmark networks are generated with fixed value *t*_1_ = 2 and *t*_2_ = 1.

**Fig 5:**
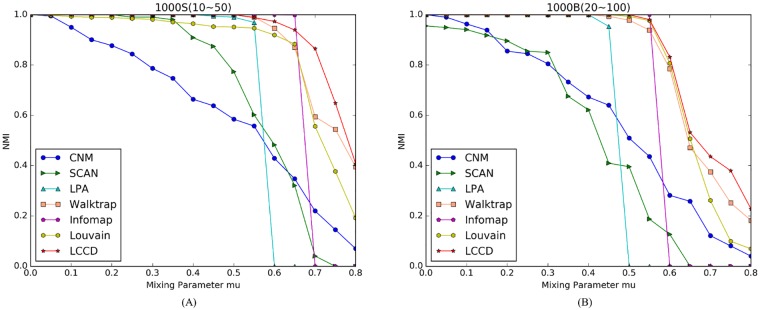
Comparison of different community detection algorithms on LFR benchmark networks with N = 1,000. (A) Benchmark networks with communities of small size. (B) Benchmark networks with communities of big size.

**Fig 6:**
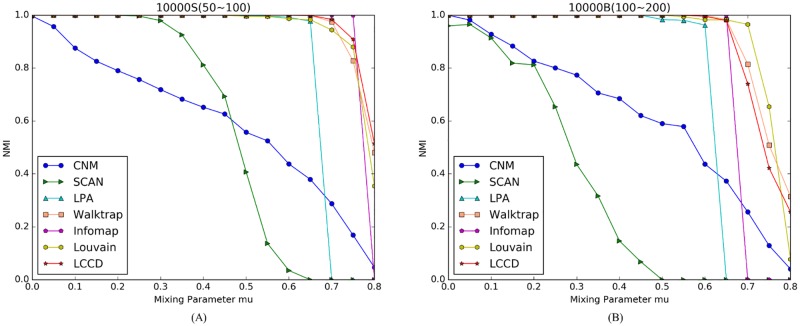
Comparison of different community detection algorithms on LFR benchmark networks with N = 10,000. (A) Benchmark networks with communities of small size. (B) Benchmark networks with communities of big size.

The NMI scores for the seven algorithms on LFR benchmark networks are presented in Figs 5 and 6. The plots correspond to two network sizes. As shown in the figures, the proposed LCCD algorithm gets NMI = 1 when *μ* < = 0.5 on the small networks with different community sizes (Fig 5), and get NMI = 1 when *μ* < = 0.6 on the large networks with two types of community sizes (Fig 6), this means that a perfect match with the original network structures. In our experiments, we find that the Infomap algorithm on the whole attains better performance when *μ* < = 0.6, compared with other algorithms, while its performance declines sharply as the mixing parameter increases. The CNM and SCAN perform worse compared to other algorithms throughout the experiments. The LPA detect the ground truth community structures both on the small network (*μ* < 0.5) and the large network (*μ* < 0.6), but its performance drops sharply when mu increases. This is because big communities are produced during label propagation when the boundary between communities is increasingly obscure.

As shown in Fig 5(A), the NMI values for Louvain are near to 1 when *μ* < 0.6, because the resolution limit of the modularity exists in networks with lots of small communities. Such situation is not obvious in following experiment, since the size of community increases gradually. There are two algorithms, i.e., Walktrap and Louvain, which achieve comparable performance to the proposed algorithm LCCD. In the small networks, all the three algorithm can identify the ground truth community structures (NMI = 1) when mixing parameter *μ* is less than 0.5, and then their performance declines, as shown in Fig 5(B). However, such trend is different in the large networks. The three algorithms detect the true community structures when *μ* is no larger than 0.65. Comparing the Fig 6(A) and 6(B), we note that Louvain gradually shows obvious advantage in the aspects of identify big communities, and achieves better performance when *μ* > 0.7. On the other hand, we find that LCCD has an advantage over other methods in finding small community structures in networks. Based on above comparative analysis, we can conclude that our algorithm works well and achieve better performance, compared to other algorithms.

#### Test on Real-world Networks

In order to further illustrate the effectivity and feasibility of the proposed method, we compare the performance of LCCD with the compared algorithms on sixteen real-world networks. These networks include social networks, such as Zachary’s karate club network and dolphin social network, and politic book network, scientist collaboration network, and biological networks. All these networks have been commonly used in community detection. The simple description of each network is as follows.

The Zachary’s Karate club network [49] reflects social interactions among the members of a karate club, which contains 34 members and 78 edges. The club ultimately was divided into two distinct groups because of a disagreement between the administrator (vertex 1) and the instructor (vertex 34), and these two groups are used as the ground truth communities in benchmark studies.

The dolphin social network was constructed based on the observations recording frequent associations between a group of 62 bottlenose dolphins over a period of 7 years from 1994 to 2001 [50]. In this network, dolphins represented as nodes have an edge with each other if they are observed together more often than expected by chance. In previous study, it is generally divided into two communities or four sub-communities in term of sex and age of dolphins.

Social network of positive sentiment [51]described the social relationships between inmates in prison, in which nodes represent people in a group, and edges represent positive sentiment directed from one group member to another, based on questionnaires. Lesmis network [52]is a coappearance network of characters in the novel “Les Miserables”, which consists of 77 nodes and 254 edges.

Political book network includes 105 nodes that represent books about US politics sold by the online bookseller Amazon.com [53]. Edges represent frequent co-purchasing of books by the same buyers, as indicated by the “customers who bought this book also bought these other books” feature on Amazon. The political viewpoints of these books are given by “liberal”, “neutral” and “conservative”, respectively, which are taken as the ground-truth in our experiment.

Word network is the adjacency network of common adjectives and nouns in the novel “David Copperfield” by Charles Dickens [54]. Nodes represent the most commonly occurring adjectives and nouns in the book. Edges connect any pair of words that occur in adjacent position in the text of the book. College football network represents the schedule of games between American college football teams during regular season [40]. In the network nodes denote the 115 teams that are divided into 12 conferences, and the edges represent 616 games.

Jazz musicians networks modeled the topology structure of the collaboration network of jazz musicians, which includes 198 bands that performed between 1912 and 1940 [55]. An edge between two bands is established if they have at least one musician in common.

Three biological networks are included. Neural network represents the neural network of C. Elegans [56]. Metabolic network represents metabolic system of C.Elegans [57]. Yeast transcription network describes transcription interactions between regulatory proteins and genes in the bacterium and the yeast [58].

Email network represents e-mail interchanges between members of the Univeristy Rovira i Virgili [59]. Polblogs network describes the political blogosphere network of hyperlinks between weblogs on US politics in 2004 [60]. Netscience network records coauthorship of scientists working on network theory and experiments [54], in which various connected components exist. Power network represents the topology of the Western States Power Grid of the United States [56]. Collaboration network covers scientific collaborations between authors’ papers submitted to General Relativity and Quantum Cosmology category [61].

The size of these networks above ranges from tens of to thousands of nodes, and the number of communities varies in different networks. Some weighted networks are transformed to unweighted ones by setting the weight of all edges as 1. Detailed information about these networks are shown in Table 2. All network dataset can be attained from webpages [62–64].

**Table 2:** The basic information of the real-world networks.

Network	*N*	*M*	〈*k*〉	*nCluster*
Karate	34	78	4.59	2
Dolphin	62	159	5.13	2
Social	67	182	4.24	21
Lesmis	77	254	6.59	6
Polbooks	105	441	8.40	3
Word	112	425	7.59	7
Football	115	613	10.66	12
Jazz	198	2,742	27.70	4
Neural	297	2,148	14.46	5
Metabolic	453	2,025	8.94	25
Yeast	688	1,078	3.13	26
Email	1,133	5,451	9.62	11
Polblogs	1,490	16,715	22.44	4
Netscience	1,589	2,742	3.45	406
Power	4,941	6,594	2.67	40
Collaboration	5,242	14,496	8.30	395

*N* and *M* represent the number of nodes and the numbers of edges in network, respectively. 〈*k*〉 denotes the average degree of the network. *nCluster* denotes the numbers of the ground truth communities in the network or the optimal number of communities with the largest modularity value.

We adopt the modularity measurement to evaluate the accuracy of algorithms on these real-world networks. Larger modularity scores indicate more cohesive community structures. The comparative results of modularity are shown in Table 3. Numbers in boldface denote the largest values of modularity in the corresponding row. In this table, we get the following observations. The LCCD algorithm performs better and obtains optimal results for over half of the 16 real-world networks (56%) in terms of modularity, compared to other algorithms. In addition, Louvain acquires best results in other 6 networks (37.5%), and CNM gets a best value only in the Word network. Higher percentage indicates more stable performance on various networks. This result shows that LCCD method illustrates the superiority of local expansion strategy and achieves better performance on real-world networks of complicated organizational structures.

**Table 3:** The comparison of modularity values on the real-world networks.

Network	CNM	SCAN	LPA	Walktrap	Infomap	Louvain	LCCD
Karate	0.3807	0.3409	0.1328	0.3532	0.4020	0.4188	**0.4197**
Dolphin	0.4955	0.2887	0.4876	0.4888	0.5247	0.5185	**0.5257**
Social	0.5565	0.4292	0.5515	0.5460	0.5697	**0.5741**	0.5702
Lesmis	0.5006	0.2258	0.5344	0.5214	0.5462	**0.5556**	0.5376
Polbooks	0.5020	0.4045	0.4874	0.5070	0.5228	0.5205	**0.5255**
Word	**0.2947**	0.1130	0	0.2162	0.0092	0.2886	0.2425
Football	0.5497	0.5143	0.6022	0.6029	0.6005	0.6046	**0.6072**
Jazz	0.4389	0.2689	0.2780	0.4384	0.2800	0.4431	**0.4529**
Neural	0.3723	0.2256	0.2090	0.3532	0.3582	**0.3876**	0.3670
Metabolic	0.4055	0.3078	0.0585	0.3487	0.4134	**0.4407**	0.3512
Yeast	0.7572	0.3109	0.7351	0.7426	0.7194	**0.7639**	0.7484
Email	0.5070	0.3017	0.0717	0.5307	0.5231	0.5426	**0.5676**
Polblogs	0.4269	0.3269	0.4258	0.4254	0.4228	0.4269	**0.4336**
Netscience	0.9551	0.8957	0.9101	0.9559	0.9303	0.9597	**0.9659**
Power	0.9335	0.5674	0.8019	0.8310	0.8161	0.9363	**0.9398**
Collaboration	0.8142	0.6945	0.7952	0.7817	0.7936	**0.8630**	0.8232

Bold number in each row denotes the best value in corresponding item.

Moreover, LCCD gets better results on all networks (100%) compared to SCAN, Walktrap, and LPA. In general, LPA has satisfying time efficiency, but its performance is far from satisfying because of the indeterminacy in label propagation. The modularity values of CNM on most networks are smaller than LCCD except on Word network. LCCD also outperforms Infomap on most networks (87.5%), except for the Lesmis and Metabolic network. This observation is in agreement with the fact that our algorithms can achieve better performance on synthetic networks shown in Figs 5 and 6. Therefore, we can conclude that the proposed LCCD is an effective and competitive method for identifying community structures.

## Conclusion

In this work, we formulate the community structure as a centralized hierarchical structure which is constituted by structural center, cores and outliers. In order to identify such structure, a novel density-based algorithm named as LCCD has been presented. The main characteristic of LCCD is that it is based on the proposed structural centrality, which takes into account local density of nodes and relative distance between clusters. With structural centrality, we have developed the algorithm for locating structural centers that can determine the number of clusters in networks automatically. This algorithm depends on finding local maximum of structural centrality, and can be combined with other clustering algorithms for community detection.

LCCD uncovers intrinsic community structures by a local expansion from the identified structural centers with a local search procedure. Such expanding process avoids the randomness of seed selection and manual choice of built-in parameters. Furthermore, by defining a border region in a community, cores and outliers can be identified in node assignment phase, which is an important feature that LCCD includes. The extensive experiments on both synthetic and real-world networks demonstrate the advantages of LCCD from three aspects. First of all, the local expanding procedure based on structural centers accelerates its convergence to optimal partition, and make the algorithm perform more steadily. Secondly, our algorithm is local expansion method for community detection, which avoids the resolution problem of modularity. Lastly, LCCD not only uncovers natural community structures effectively, but also identifies hierarchical structure of a community. Some meaningful extensions can be made to LCCD in the future. With some improvement, it can be used for uncovering overlapping community.

## Supporting Information

S1 Fig A schematic example to illustrate the idea of the method. (A) The Zachary’s karate club network with two clusters; (B) The decision graph for the nodes in the network.
(TIF)

S2 Fig Location of structural centers on synthetic LFR network with 1,000 nodes and 9 ground-truth communities. (A) The node distribution in the decision graph; (B) The plot of structural centrality sorted in decreasing order as a function of node number for the network.
(TIF)

S3 Fig An illustration of node assignment with an abstract graph. (TIF)

S4 Fig Comparison on GN benchmarks. (TIF)

S5 Fig Comparison of different community detection algorithms on LFR benchmark networks with N = 1,000. (A) Benchmark networks with communities of small size; (B) Benchmark networks with communities of big size.
(TIF)

S6 Fig Comparison of different community detection algorithms on LFR benchmark networks with N = 10,000. (A) Benchmark networks with communities of small size; (B) Benchmark networks with communities of big size.
(TIF)

S1 Table The main parameters of the generated benchmark networks. *N* represents number of nodes, 〈*k*〉 denotes average degree of nodes. All benchmark networks are generated with fixed value *t*_1_ = 2 and *t*_2_ = 1.
(DOCX)

S2 Table The basic information of the real-world networks. *N* and *M* represent the number of nodes and the number of edges in network, respectively. 〈*k*〉 denotes the average degree of the network. *nCluster* denotes the numbers of the ground truth communities in the network or the optimal number of communities with the largest modularity value.
(DOCX)

S3 Table The comparison of modularity values on the real-world networks. Bold number in each row denotes the best value in corresponding item.
(DOCX)

S1 Appendix Independence on the cutoff distance. (PDF)
