# Supplementary figures and images for: Locating Structural Centers: A Density-Based Clustering Method for Community Detection
Source: PLoS One. 2017 Jan 3;12(1):e0169355. doi: 10.1371/journal.pone.0169355 (PMC5207651; doi:10.1371/journal.pone.0169355)

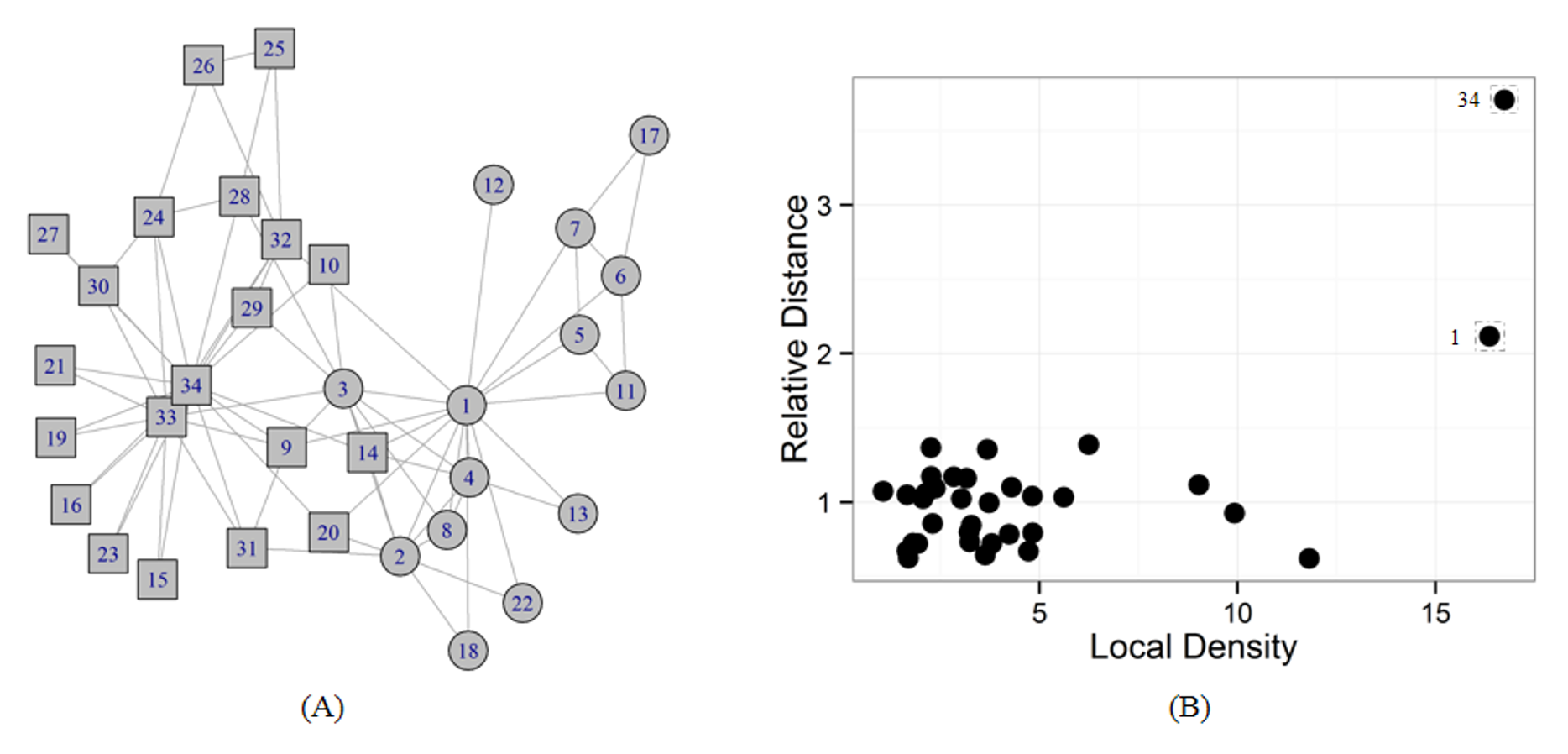

Supplement: S1 Fig — (A) The Zachary’s karate club network with two clusters; (B) The decision graph for the nodes in the network. (TIF) [file pone.0169355.s001.tif]

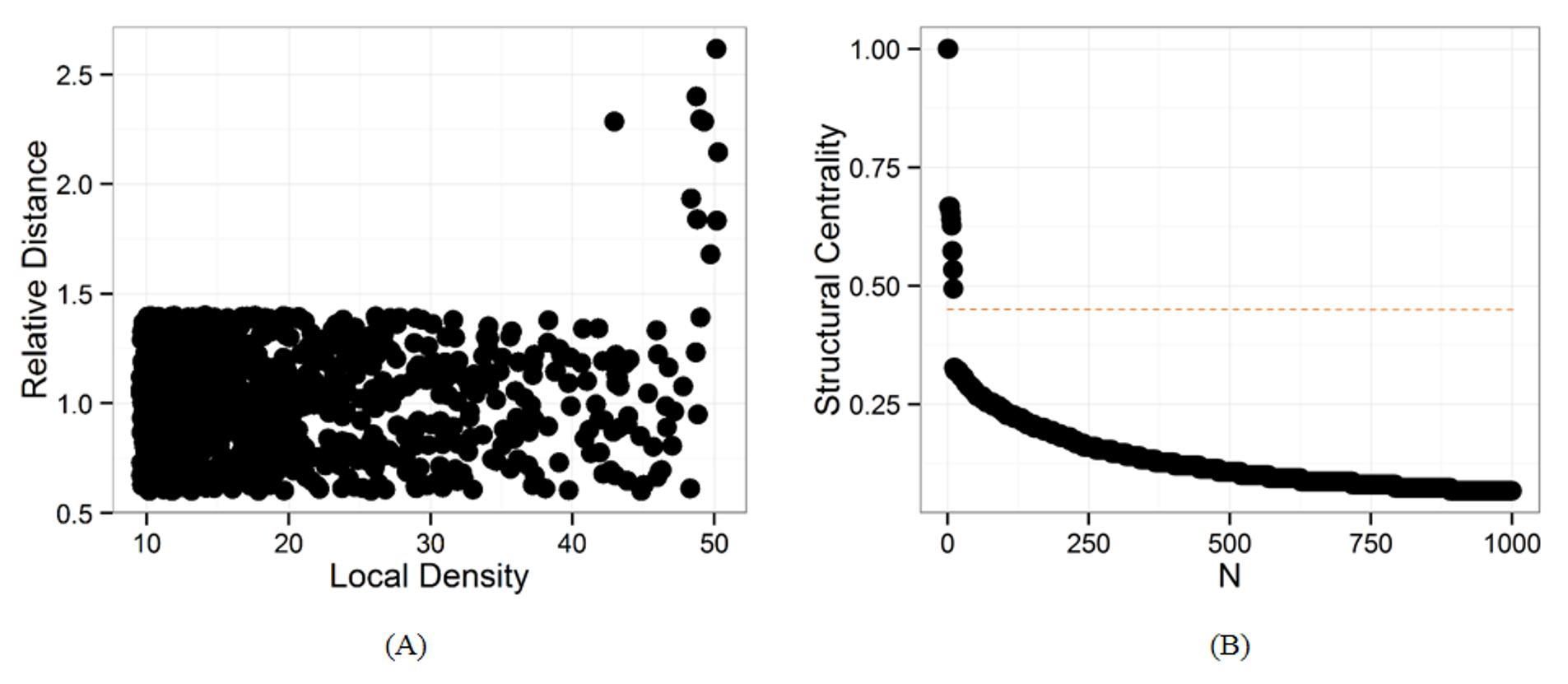

Supplement: S2 Fig — (A) The node distribution in the decision graph; (B) The plot of structural centrality sorted in decreasing order as a function of node number for the network. (TIF) [file pone.0169355.s002.tif]

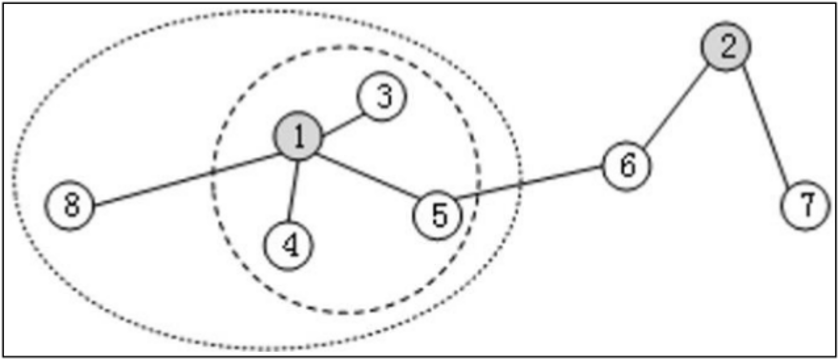

Supplement: S3 Fig — (TIF) [file pone.0169355.s003.tif]

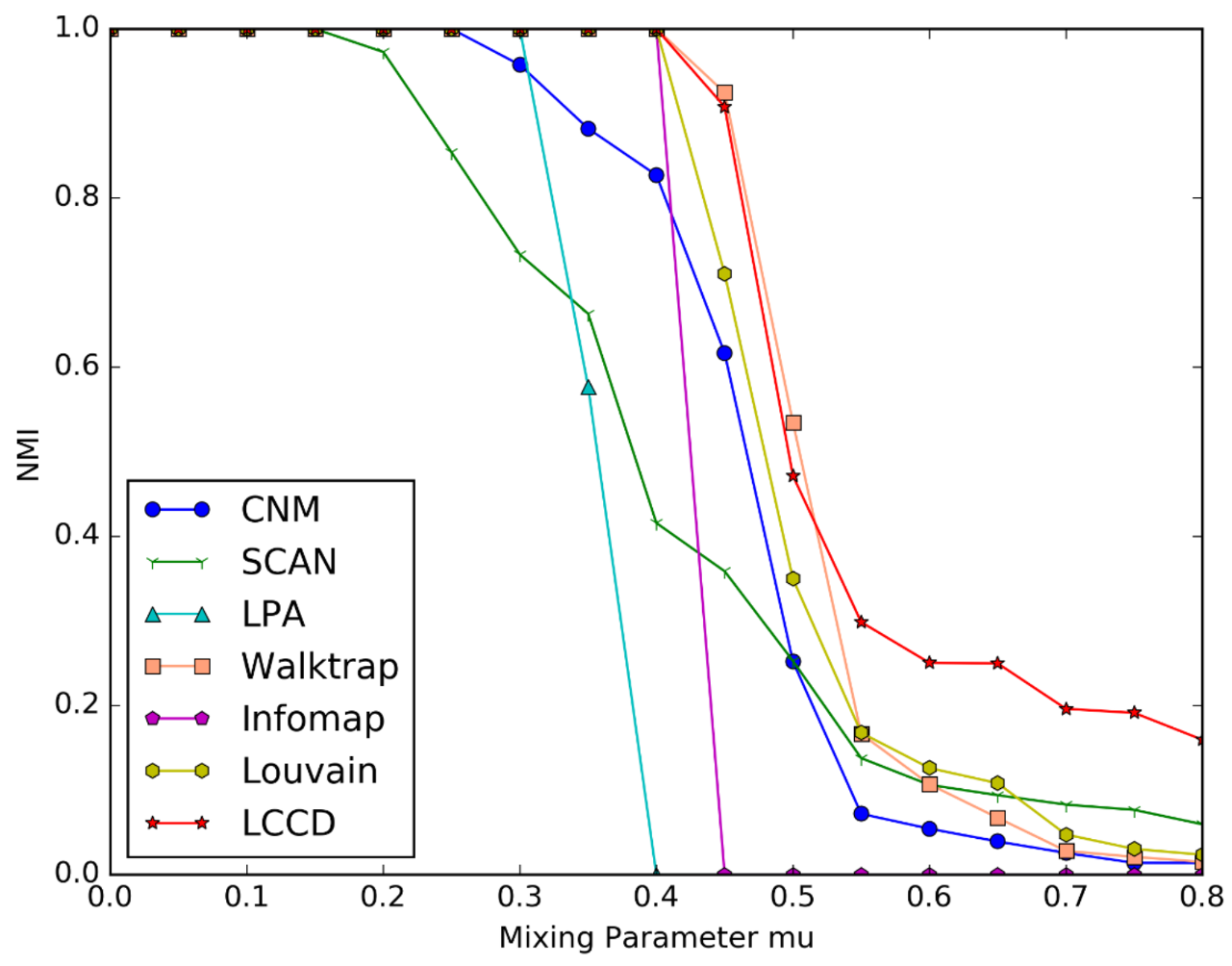

Supplement: S4 Fig — (TIF) [file pone.0169355.s004.tif]

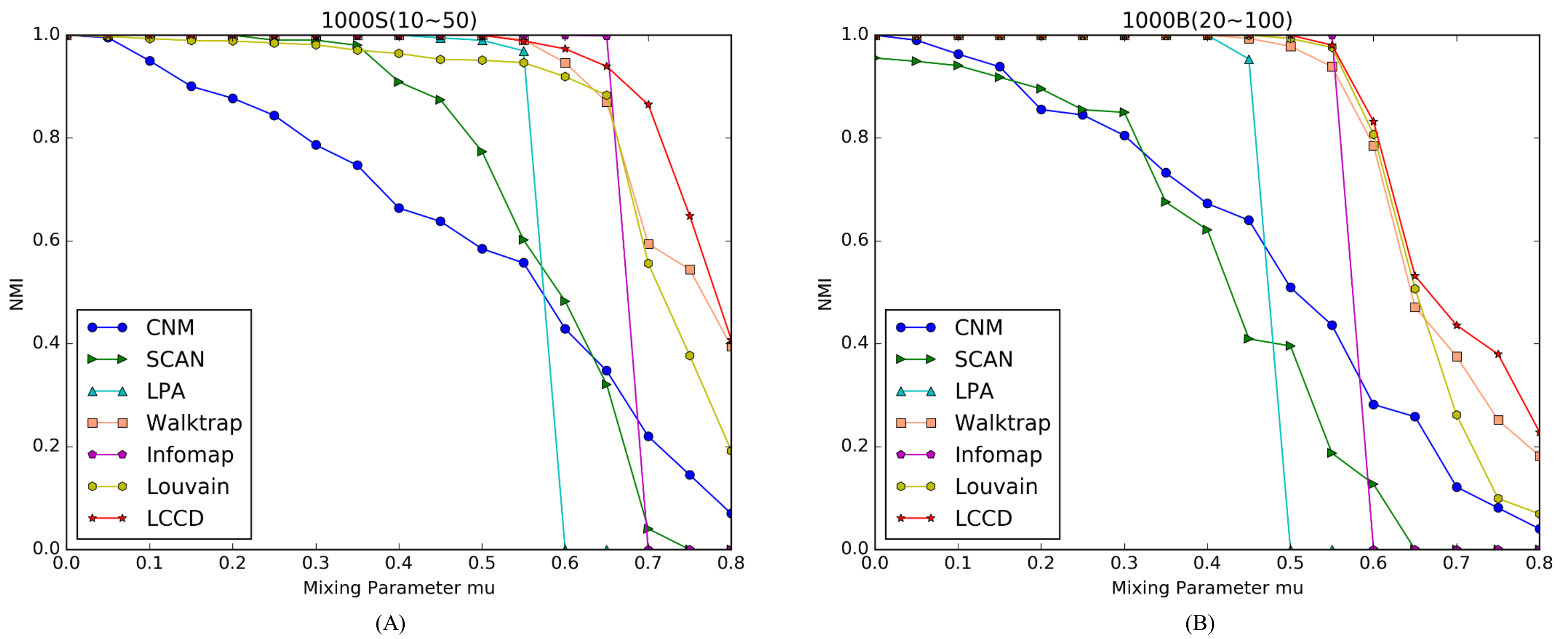

Supplement: S5 Fig — (A) Benchmark networks with communities of small size; (B) Benchmark networks with communities of big size. (TIF) [file pone.0169355.s005.tif]

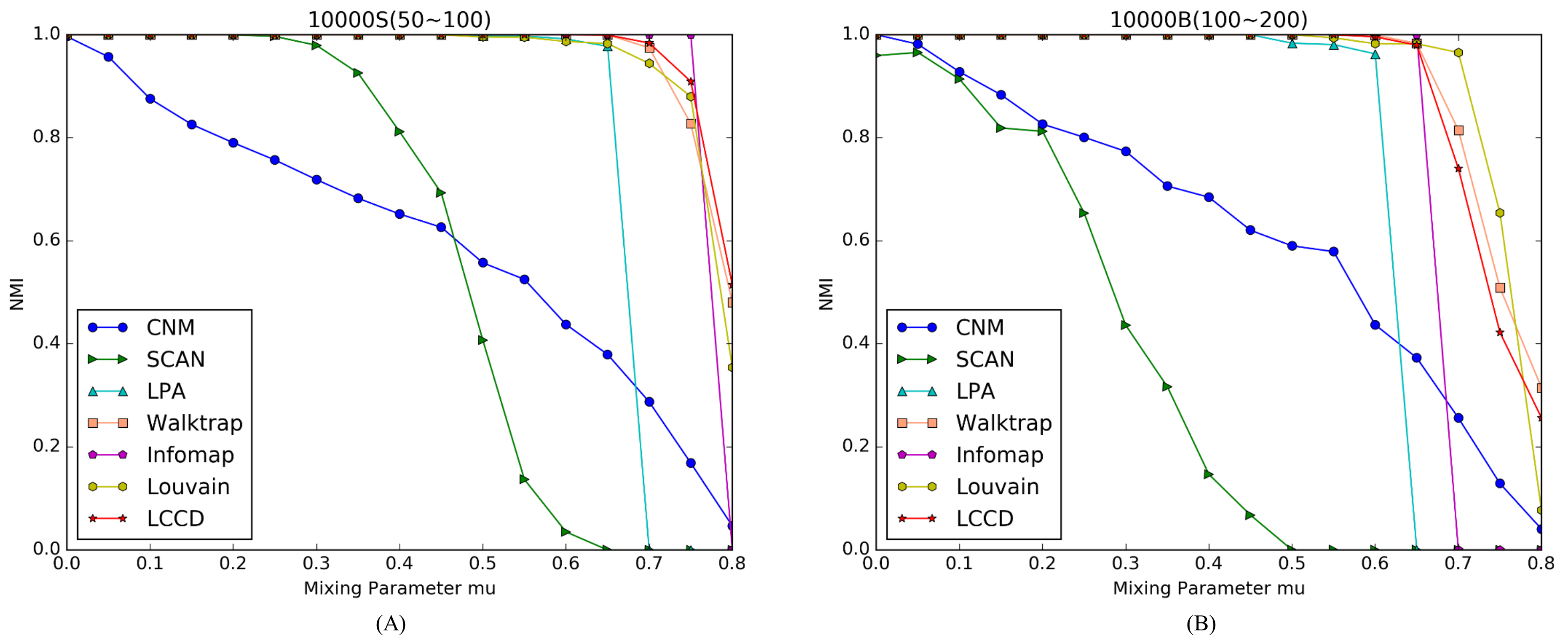

Supplement: S6 Fig — (A) Benchmark networks with communities of small size; (B) Benchmark networks with communities of big size. (TIF) [file pone.0169355.s006.tif]
