# Supplementary material for: Locating Structural Centers: A Density-Based Clustering Method for Community Detection
Source: PLoS One. 2017 Jan 3;12(1):e0169355. doi: 10.1371/journal.pone.0169355 (PMC5207651; doi:10.1371/journal.pone.0169355)
Supplement: S1 Table — N represents number of nodes, 〈k〉 denotes average degree of nodes. All benchmark networks are generated with fixed value t1 = 2 and t2 = 1. (DOCX) [file pone.0169355.s007.docx]

**Table 1.** **The main parameters of the generated benchmark networks.**

| **Network** | ***N*** | ***k*** | ***maxk*** | ***minc*** | ***maxc*** |
| --- | --- | --- | --- | --- | --- |
| 1000S | 1,000 | 20 | 50 | 10 | 50 |
| 1000B | 1,000 | 20 | 50 | 20 | 100 |
| 10000S | 10,000 | 40 | 100 | 50 | 100 |
| 10000B | 10,000 | 40 | 100 | 100 | 200 |

*N* represents number of nodes; *k* denotes average degree of nodes; *maxk* represents the maximum degree of nodes; *minc* denotes the minimum community size, and *maxc* the maximum one. All benchmark networks are generated with fixed value $t_{1}=2$ and $t_{2}=1$.
