# Supplementary material for: Locating Structural Centers: A Density-Based Clustering Method for Community Detection
Source: PLoS One. 2017 Jan 3;12(1):e0169355. doi: 10.1371/journal.pone.0169355 (PMC5207651; doi:10.1371/journal.pone.0169355)
Supplement: S2 Table — N and M represent the number of nodes and the number of edges in network, respectively. 〈k〉 denotes the average degree of the network. nCluster denotes the numbers of the ground truth communities in the network or the optimal number of communities with the largest modularity value. (DOCX) [file pone.0169355.s008.docx]

**Table 2**. **The basic information of the real-world networks.**

| **Network** | ***N*** | ***M*** | ***<k>*** | ***nCluster*** |
| --- | --- | --- | --- | --- |
| Karate | 34 | 78 | 4.59 | 2 |
| Dolphin | 62 | 159 | 5.13 | 2 |
| Social | 67 | 182 | 4.24 | 21 |
| Lesmis | 77 | 254 | 6.59 | 6 |
| Polbooks | 105 | 441 | 8.40 | 3 |
| Word | 112 | 425 | 7.59 | 7 |
| Football | 115 | 613 | 10.66 | 12 |
| Jazz | 198 | 2,742 | 27.70 | 4 |
| Neural | 297 | 2.148 | 14.46 | 5 |
| Metabolic | 453 | 2,025 | 8.94 | 25 |
| Yeast | 688 | 1,078 | 3.13 | 26 |
| Email | 1,133 | 5.451 | 9.62 | 11 |
| Polblogs | 1,490 | 16,715 | 22.44 | 4 |
| Netscience | 1,589 | 2,742 | 3.45 | 406 |
| Power | 4,941 | 6,594 | 2.67 | 40 |
| Collaboration | 5,242 | 14,496 | 8.30 | 395 |

In the table, *N* and *M* represent the number of nodes and the number of edges in network, respectively. *<k>* denotes the average degree of the network. *nCluster* denotes the numbers of the ground truth communities in the network or the optimal number of communities with the largest modularity value.
