# Supplementary material for: Locating Structural Centers: A Density-Based Clustering Method for Community Detection
Source: PLoS One. 2017 Jan 3;12(1):e0169355. doi: 10.1371/journal.pone.0169355 (PMC5207651; doi:10.1371/journal.pone.0169355)
Supplement: S3 Table — Bold number in each row denotes the best value in corresponding item. (DOCX) [file pone.0169355.s009.docx]

**Table 3. The comparison of modularity values on the real-world networks.**

| **Network** | **CNM** | **SCAN** | **LPA** | **Walktrap** | **Infomap** | **Louvain** | **LCCD** |
| --- | --- | --- | --- | --- | --- | --- | --- |
| Karate | 0.3807 | 0.3409 | 0.1328 | 0.3532 | 0.4020 | 0.4188 | **0.4197** |
| Dolphin | 0.4955 | 0.2887 | 0.4876 | 0.4888 | 0.5247 | 0.5185 | **0.5257** |
| Social | 0.5006 | 0.2258 | 0.5344 | 0.5214 | 0.5462 | **0.5556** | 0.5376 |
| Lesmis | 0.5565 | 0.4292 | 0.5515 | 0.5460 | 0.5697 | **0.5741** | 0.5702 |
| Polbooks | 0.5020 | 0.4045 | 0.4874 | 0.5070 | 0.5228 | 0.5205 | **0.5255** |
| Word | **0.2947** | 0.1130 | 0 | 0.2162 | 0.0092 | 0.2886 | 0.2425 |
| Football | 0.5497 | 0.5143 | 0.6022 | 0.6029 | 0.6005 | 0.6046 | **0.6072** |
| Jazz | 0.4389 | 0.2689 | 0.2780 | 0.4384 | 0.2800 | 0.4431 | **0.4529** |
| Neural | 0.3723 | 0.2256 | 0.2090 | 0.3532 | 0.3582 | **0.3876** | 0.3670 |
| Metabolic | 0.4055 | 0.3078 | 0.0585 | 0.3487 | 0.4134 | **0.4407** | 0.3512 |
| Yeast | 0.7572 | 0.3109 | 0.7351 | 0.7426 | 0.7194 | **0.7639** | 0.7484 |
| Email | 0.5070 0.3017 | 0.3017 | 0.0717 | 0.5307 | 0.5231 | 0.5426 | **0.5676** |
| Polblogs | 0.4269 | 0.3269 | 0.4258 | 0.4254 | 0.4228 | 0.4269 | **0.4336** |
| Netscience | 0.9551 | 0.8957 | 0.9101 | 0.9559 | 0.9303 | 0.9597 | **0.9659** |
| Power | 0.9335 | 0.5674 | 0.8019 | 0.8310 | 0.8161 | 0.9363 | **0.9398** |
| Collaboration | 0.8142 | 0.6945 | 0.7952 | 0.7817 | 0.7936 | **0.8630** | 0.8232 |

Bold number in each row denotes the best value in corresponding item.
