# Supplementary material for: Locating Structural Centers: A Density-Based Clustering Method for Community Detection
Source: PLoS One. 2017 Jan 3;12(1):e0169355. doi: 10.1371/journal.pone.0169355 (PMC5207651; doi:10.1371/journal.pone.0169355)
Supplement: S1 Appendix — (PDF) [file pone.0169355.s010.pdf]

Appendix. Independence on the cutoff distance.

The choice of the cutoff distance d_c where the local density of a node is computed may affect structural centers identified by structural centrality. This means, in principle, that we cannot rely on a specific choice of cutoff distance. However, we have found that our algorithm is sensitive only to the relative magnitude of node density, which implies that the results are robust with respect to choice of d_c. In our experiments, we observe that d_c is restricted to several values which are much smaller than the network diameter under study. In most case, cutoff distance d_c gets a value of 1. We have collected cutoff values for some well-known real-world networks that also are used in our experiments, as shown in Table 1.

Table 1. The cutoff distance d_c for the real-world network in the experiments.

Network	N	M	D	d_c	Network	N	M	D	d_c
Karate	34	78	5	1	Neural	297	2,148	5	1
Dolphin	62	159	8	1	Metabolic	453	2,025	7	2
Social	67	182	7	1	Yeast	688	1,078	15	2
Lesmis	77	254	5	1	Email	1,133	5,451	8	2
Polbooks	105	441	7	1	Polblogs	1,490	16,715	8	2
Word	112	425	5	1	Netscience	1,589	2,742	17	5
Football	115	4	1	1	Power	4,941	6,594	46	6
Jazz	198	2,742	6	1	Collaboration	5,242	14,496	17	3

N and M represent the number of nodes and the number of edges in network, respectively. D denotes the diameter of the network. d_c denotes the cutoff distance for the network. Detail information on the real-world networks is included in the manuscript.

We also have investigated the choice of cutoff distance on LFR benchmark networks. The LFR networks have obvious statistic properties of real world networks. Specifically, The node degree and community size of the network both follow the power-law distribution and have scale-free properties. We have analyzed the cutoff distance on a variety of benchmark networks with varying mixing parameters and different community scale. The statistic results are shown in Table 2. From this table, we can conclude that for all benchmark networks the cutoff distance is much small than the diameter. And more importantly, the values of cutoff distance equal 1 in most case except for the network with big community when $\mu = 0.7$. Moreover, we find that varying cutoff distance produces mutual consistent results, which implies the algorithm is robust to the choice of cutoff distance. For the exception in the synthetic networks, we test the algorithm by varying cutoff distance for the benchmark network with $d_c = 2$ in Table 2, the results are shown in Figure. 1. We can observe that ten structural centers are identified in both two panels, which illustrates that consistent results are achieved.

Table 2. The cutoff distance d_c for the synthetic network in the experiments.

LFR		0	0.1	0.2	0.3	0.4	0.5	0.6	0.7	0.8
1000B	D	21	5	5	4	4	4	4	10	4
	d_c	1	1	1	1	1	1	1	2	1
1000S	D	9	6	5	5	4	4	4	4	4
	d_c	1	1	1	1	1	1	1	1	1

LFR benchmark networks are generated with parameters $N = 1000$, $k = 20$, $k_{max} = 50$, t_1=2, t_2=1 and varying mixing parameters. 1000B represents a set of LFR benchmark networks with big communities ranging 50 from 100.1000S represents a set of LFR benchmark networks with small communities ranging 20 from 50. D denotes the diameter of the network. d_c denotes the cutoff distance for the network.

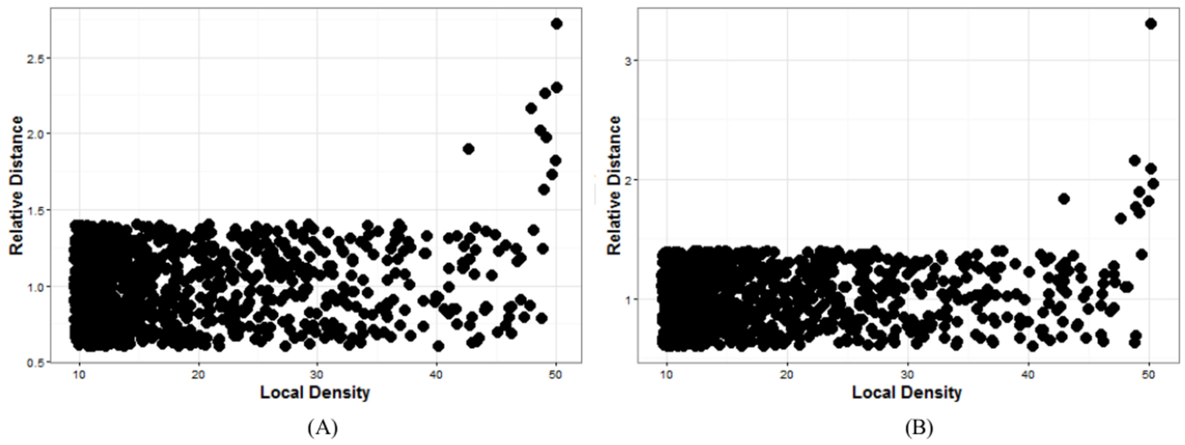

Figure 1. Comparison of the assignation for different values of cutoff distance d_c for the benchmark network with $d_c = 2$ in Table 2. Although the value of d_c varies, consistent results are produced in the test. (A) The identified structural centers by the algorithm with $d_c = 1$; (B) The identified structural centers by the algorithm with $d_c = 2$.
